# Predictive immunonutritional biomarkers for the severity and recovery of sudden sensorineural hearing loss: a prospective clinical study

**DOI:** 10.3389/fneur.2025.1542386

**Published:** 2025-08-22

**Authors:** Xu Zhang, Weixun Guo, Bing Guan, Chunping Yang

**Affiliations:** ^1^Department of Otorhinolaryngology Head and Neck Surgery, The Second Affiliated Hospital, Jiangxi Medical College, Nanchang University, Nanchang, Jiangxi, China; ^2^Department of Neurology, Xuyi People's Hospital, Huai'an, China; ^3^Department of Otolaryngology-Head and Neck Surgery, Northern Jiangsu People's Hospital Affiliated to Yangzhou University, Yangzhou, Jiangsu, China

**Keywords:** PNI, SII, SSNHL, immune-inflammation, immune-nutritional status

## Abstract

**Background:**

Systemic immune-inflammatory index (SII) and prognostic nutritional index (PNI) are known to predict the severity and prognosis of various diseases. However, their role in sudden sensorineural hearing loss (SSNHL) is unclear.

**Methods:**

This study collected 100 patients with SSNHL and 100 healthy volunteers. According to the severity, type, prognosis and SII and PNI levels of SSNHL, we used the Spearman linear correlation method to conduct correlation analysis. At the same time, we constructed logistic regression analysis to explore the predictive value of PNI and SII on the prognosis of SSNHL patients, and used ROC curves to verify the prognostic model. Cohen’s d values were calculated for select significantly different parameters to assess effect sizes.

**Results:**

Compared with control group, PNI levels were significantly lowered in SSNHL patients, while SII levels were significantly higher. And a significant correlation was observed between the two variables (*R* = -0.437, *p* < 0.001). At the same time, compared with patients with mild and moderate SSNHL, patients with severe SSNHL had the lowest PNI levels and the highest SII levels. PNI showed a negative correlation with hearing loss severity once the proper categorization of severity was taken into account, whereas SII was positively correlated with severity. After adjusting for potential confounders, both high SII and low PNI were independently associated with worse prognosis in SSNHL. Effect size analysis (Cohen’s d) revealed moderate practical significance in the differences in PNI levels between groups.

**Conclusion:**

In patients with SSNHL, PNI levels were significantly lowered, while SII levels were significantly higher. Furthermore, a negative correlation was observed between these two indicators. A negative correlation between PNI and SSNHL severity and a positive correlation between SII and severity were observed. These findings suggest that PNI and SII could serve as potential biomarkers for predicting SSNHL prognosis.

## Introduction

Sudden sensorineural hearing loss (SSNHL) is a common otologic emergency, characterized by rapid onset sensorineural hearing loss of ≥30 dB at three contiguous frequencies within 72 h (1). The incidence of SSNHL is about 16–160 cases per 100,000 people per year. 70% of patients can have varying degrees of hearing improvement within 6 months, but about 30% of patients still have persistent hearing loss (2). SSNHL significantly affects the daily functioning and quality of life of patients, necessitating prompt and serious attention. Timely intervention is crucial for individuals with SSNHL. Administering effective treatment within 2 weeks of onset can result in a recovery rate of approximately 60–80%. Conversely, if diagnosis and treatment are delayed beyond 1 month, the recovery rate diminishes substantially to only 10–20% (3). The etiology of SSNHL remains uncertain. Current hypotheses propose that the onset of the condition may be linked to factors including viral infections, vascular disorders, autoimmune diseases, and other related variables (1). Considering the challenges in preventing and treating SSNHL and the difficulty in achieving optimal treatment outcomes, investigating the clinical characteristics and associated risk factors of SSNHL is of paramount importance for enhancing the early diagnosis rate in affected patients.

Current prognostic indicators for SSNHL include the patient’s age, the severity of initial hearing loss, the time of treatment initiation, and early signs of hearing recovery (4). However, these factors are predominantly based on clinical observations, lack a robust biological foundation, exhibit considerable limitations, and provide limited utility in the diagnosis and prognosis of SSNHL. In recent years, the hypothesis of pathological activation of cellular stress pathways has garnered significant attention (5), highlighting the role of immune inflammation in SSNHL. Consequently, the body’s immune and nutritional status has been recognized as crucial to auditory health (6).

In this context, the systemic immune-inflammatory index (SII) and the prognostic nutritional index (PNI) have demonstrated their distinct significance as potential predictive biomarkers. The SII, which is calculated using neutrophil, platelet, and lymphocyte counts, provides insight into the body’s immune-inflammatory status (7).

Elevated SII value is frequently associated with a more pronounced inflammatory response. Furthermore, recent studies indicate that SII serves as a significant prognostic indicator for a range of diseases, including cardiovascular disorders and malignancies (8, 9). A study showed that high SII value was generally associated with a worse prognosis in SSNHL (10). Simultaneously, the PNI is determined by evaluating serum albumin levels and lymphocyte counts, serving as an indicator of patients’ nutritional status and immune function (11). Low PNI values frequently suggest malnutrition and compromised immune function, and PNI has been demonstrated to be a significant prognostic factor in various diseases (12, 13). In short, the aforementioned indicators provide a comprehensive assessment of the patient’s systemic inflammatory state and nutritional status. By integrating the SII and the PNI, clinicians can attain a more nuanced understanding of the physiological condition of patients with SSNHL, thereby facilitating a more precise prognostic evaluation.

In this prospective study, we investigated baseline SII and PNI levels in SSNHL, evaluated their associations with hearing severity, and explored their prognostic utility for hearing recovery. Additionally, to determine whether observed statistical differences in PNI values have practical relevance, we performed an effect size analysis (Cohen’s d). By clarifying the relationship between these immune-nutritional indicators and SSNHL progression, our goal is to facilitate early diagnosis, guide targeted therapies, and improve patient outcomes.

## Methods

### Study subjects

The study cohort comprised patients admitted to the Department of Otolaryngology and Head and Neck Surgery at Yangzhou University School of Clinical Medicine between April 2024 to December 2024. The cohort included 100 SSNHL patients admitted during this period, along with 100 healthy volunteers who were recruited concurrently. Informed consent was obtained from all participating patients, their families, and healthy volunteers. The study protocol received approval from the hospital’s Ethics Committee (approval number: 2023ky051). Among the cohort, the criteria for inclusion in the SSNHL group were: (1) Age ≥18 years, unilateral onset of sensorineural hearing loss of ≥30 dB at three contiguous frequencies, occurring within 72 h; (2) unknown etiology, including systemic or local factors; (3) Possible symptoms such as tinnitus, ear fullness, or paresthesia around the ears; (4) Possible accompanying dizziness, nausea, or vomiting; (5) No prior use of steroid medication. The exclusion criteria were as follows: (1) failure to meet the inclusion criteria; (2) organic lesions of the middle ear; and (3) coexisting conditions known to cause hearing loss, including acute or chronic renal failure, diabetes, hypertension, hyperlipidemia, coronary heart disease, chronic liver disease, lung disease, infectious disease, immune disease, and any ear disease (such as chronic otitis media, otosclerosis, history of ear trauma, and Meniere’s disease), vestibular schwannoma, or traumatic lymphatic fistula were exclusion criteria.

### Data processing

All patients underwent general clinical interviews, laboratory tests, otoendoscopy, and audiological examinations. In addition, brain MRI was performed to exclude cochlear space-occupying diseases.

According to the efficacy evaluation criteria of *Guideline of diagnosis and treatment of sudden deafness (2015, China)* (14), the hearing assessment is mainly determined by the pure tone average (PTA) from 125 Hz to 8 kHz. The pure tone average (PTA-4) was determined by calculating the average hearing threshold at frequencies of 250, 500, 1,000, and 2,000 Hz. If the PTA is less than 25 dB HL, the hearing is defined as normal, if the PTA is 26 dB HL but less than 40 dB HL, the severity of hearing loss is defined as “mild,” if the PTA is greater than 41 dB HL but less than 60 dB HL, it is “moderate,” if the PTA is greater than 61 dB HL but less than 80 dB HL, it is “severe,” and if the PTA is greater than 81 dB HL, it is “very severe.” “Mild” and “moderate” are classified as “mild to moderate,” “severe” and “extremely severe” are classified as “severe to profound.”

*Clinical characteristics included*: Gender, age, degree of hearing loss, PTA before and after treatment, side of SSNHL, vertigo (yes/no), tinnitus (yes/no), treatment time, blood pressure (systolic and diastolic) at admission, and BMI.

*Laboratory tests*: All patients had blood drawn in the morning after fasting for 8 h, including glycosylated hemoglobin A1c (HbA1C), Creatinine (Cr), albumin (Alb), aspartate aminotransferase (AST), alanine aminotransferase (ALT), total bilirubin (TBIL), indirect bilirubin (IBIL), triglycerides (TG), high-density lipoprotein (HDL), low-density lipoprotein (LDL), total cholesterol (TC), Fibrinogen (FIB), Neutrophils (NE), WBC, lymphocyte (Lym), platelet (Plt), Platelet_volume, and D-D dimer (DD). Measurement of SII: platelet count × neutrophil count/lymphocyte count; measurement of PNI: 10 × serum albumin (g/dL) + 0.005 × total lymphocyte count (per mm).

After admission, all patients received comprehensive treatment according to *the Guidelines for the Diagnosis and Treatment of Sudden Deafness (2015, China)*, including systemic steroid therapy, with an initial dose of 80 mg of intravenous methylprednisolone, neurotrophic drugs, and intratympanic injection of dexamethasone once every other day for a total of three times. Patients with “severe” and “very severe” hearing loss were additionally treated with the fibrinogen-lowering drug batroxobin, with an initial dose of 10 BU and subsequent administration of 5 BU. Serum fibrinogen levels were monitored before each use to confirm that it was higher than 1 g/L to avoid bleeding and other related side effects. It should be emphasized that we define the course of treatment here as the duration of systemic steroids.

Follow-up hearing assessment was performed on each patient after treatment. The prognostic outcome of SSNHL was judged based on the hearing threshold of the impaired frequency by the PTA after treatment. If the PTA of the impaired frequency improved by at least 15 dB, or the impaired ear reached the same level as the normal or unaffected patient’s ear, the treatment was considered effective. If the PTA of the impaired frequency improved by less than 15 dB, the treatment was considered ineffective.

### Statistical analysis methods

Quantitative data were expressed as x̄ ± s, non-normal data were expressed as M (*Q_1_*, *Q_3_*); (M in the tables represents the median, Q1 the 25th percentile, and Q3 the 75th percentile.) qualitative data were expressed as constituent ratio (%). Kolmogorov–Smirnov test was used for normality analysis. Data were normally distributed and had equal variances. Two independent sample t test was used to test the difference between groups. Mann–Whitney U test was used when data did not follow normal distribution. PNI were normally distributed (*p* = 0.20), and two independent sample T test was used. The remaining variables did not follow normal distribution (*p* < 0.05), and U test was used to test the difference between groups. Spearman linear correlation method was used to evaluate the relationship between PNI, SII and hearing loss and hearing recovery. Binary logistics regression was constructed to explore the relationship between PNI, SII and the prognosis of SSNHL, and ROC curve was used to analyze the prediction effect of prognostic model. We used SPSS 26.0 software for statistical analysis and GraphPad prism8.0 for drawing. Test level *α* = 0.05.

## Results

### Baseline characteristics of study participants

This study included a total of 100 patients diagnosed with SSNHL and 100 healthy participants serving as the control group. The SSNHL cohort comprised 50 males (50%) and 50 females (50%). The median age of the SSNHL patients was 45.50 years (35.00 to 60.75). In comparison, the median age of the healthy control participants was 48.50 years (42.00 to 57.75). There was no statistical significance in the age difference between the two groups (*p* > 0.05). Albumin was significantly lower, and neutrophil and platelet counts were significantly higher in SSNHL patients vs. controls (*p* < 0.001). Consequently, the SII was higher and PNI was lower in SSNHL than in controls (*p* < 0.001).

Cohen’s d for PNI comparing SSNHL (49.72 ± 5.79) vs. controls (54.11 ± 4.91) was 0.82, indicating a moderate to large effect size supporting a meaningful difference. Full details are shown in Table 1.

**Table 1:** Clinical characteristics of SSNHL group and control group.

Variables	SSNHL group	Control group	*p*-value
	(*n* = 100)	(*n* = 100)	
Demographics			
Age (years)	45.50 (35.00, 60.75)	48.50 (42.00, 57.75)	0.347
BMI (kg/m^2^)	23.68 (22.10, 24.79)	22.87 (21.28, 24.99)	0.076
Gender (%)			
Male	50 (50%)	52 (52%)	0.777
Female	50 (50%)	48 (48%)	
Clinical and metabolic variables			
SBP (mmHg)	121.00 (112.25, 128.00)	122.50 (118.00, 128.00)	0.282
DBP (mmHg)	75.50 (71.00, 80.00)	78.00 (72.25, 80.00)	0.110
HDL (mg/L FEU)	1.27 (1.07, 1.61)	1.36 (1.05, 1.79)	0.309
LDL (mmol/L)	2.75 (2.38, 3.74)	3.06 (2.62, 3.58)	0.274
HbA1C (mmol/L)	5.70 (5.40, 6.20)	5.60 (5.40, 6.10)	0.437
DD (mg/L FEU)	0.21 (0.13, 0.23)	0.21 (0.14, 0.23)	0.514
TBil (mg/dL)	7.95 (6.40, 16.40)	8.95 (6.5, 16.3)	0.368
Indirect_bilirubin (mg/dL)	7.90 (4.30, 13.60)	6.20 (4.23, 11.20)	0.215
Platelet_volume (×10^9^/L)	11.35 (10.2, 12.48)	11.40 (10.20, 12.50)	0.607
Creatinine (μmol/L)	66.00 (55.00, 80.00)	68.00 (58.25, 82.25)	0.537
AST (U/L)	18.00 (16.00, 21.70)	18.00 (15.00, 22.00)	0.449
ALT (U/L)	21.00 (14.00, 28.00)	18.00 (11.00, 27.00)	0.134
TG (umol/L)	0.62 (0.543, 0.96)	0.69 (0.57, 1.05)	0.163
TC (mmol/L)	4.22 (3.88, 5.12)	4.47 (4.01, 5.17)	0.364
FIB (g/L)	2.95 (2.50, 3.58)	2.92 (2.54, 3.42)	0.502
WBC (×10^9^/L)	8.36 (6.95, 9.87)	7.87 (6.56, 9.76)	0.426
ALB (g/L)	43.00 (38.00, 45.00)	44.00 (42.00, 47.00)	<0.001
NE (×10^9^/L)	6.16 (4.07, 7.87)	4.11 (2.63, 4.57)	<0.001
LYM (×10^9^/L)	1.59 (1.20, 2.07)	1.50 (1.32, 2.45)	0.373
PLT (×10^9^/L)	257.75 (208.28, 283.67)	200.13 (176.92, 240.50)	<0.001
SII score (×10^9^/L)	854.27 (650.78, 1071.17)	376.09 (277.50, 520.49)	<0.001
PNI score (%)	49.72 ± 5.79	54.11 ± 4.91	<0.001
PTA (dBHL)	56.50 (42.00, 71.00)	-	-
Post-treatment PTA (dBHL)	48.00 (30.00, 68.00)	-	-

SBP, admission systolic blood pressure; DBP, admission diastolic blood pressure; BMI, body mass index; HbA1c, glycosylated hemoglobin A1c; Cr, Creatinine, DD, D-D dimer; TC, total cholesterol; TG, triglycerides; HDL, high-density lipoprotein; LDL, low-density lipoprotein; FIB, Fibrinogen; WBC, white blood cell; ALB, albumin; AST, aspartate aminotransferase; ALT, alanine aminotransferase; TBIL, total bilirubin; IBIL, indirect bilirubin; FIB, fibrinogen; NE, Neutrophils; LYM, lymphocyte; PLT, platelet; PTA, pure-tone average; SSNHL, sudden sensorineural hearing loss.

Based on hearing recovery after treatment, patients were divided into “effective” (*n* = 45) and “ineffective” (*n* = 55) groups. Baseline demographics showed no statistical differences in age, sex, BMI, and other laboratory markers (all *p* > 0.05). However, SII was significantly higher and PNI was significantly lower in the ineffective group vs. effective group (both *p* < 0.001). Cohen’s d for PNI between effective (53.32 ± 6.28) and ineffective (53.32 ± 6.28) groups was 0.76, again reflecting a meaningful difference. Detailed results are in Table 2.

**Table 2:** Baseline characteristics of participants in effective group or ineffective group.

Variables	Overall effective group	Ineffective group	*p*
	(*n* = 45)	(*n* = 55)	
Age (years)	40.00 (28.00, 52.00)	42.00 (34.00, 61.00)	0.091
Gender (%)	25 (50%)	25 (50%)	0.351
BMI (kg/m^2^)	23.42 (21.67, 27.88)	24.54 (23.39, 25.73)	0.051
SBP (mmHg)	125.00 (117.00, 127.40)	123.00 (108.00, 132.80)	0.152
DBP (mmHg)	73.00 (69.00, 81.50)	77.00 (72.00, 80.00)	0.221
Affected side (%)	23 (45.10%)	28 (54.90%)	0.984
Tinnitus (%)	38 (44.71%)	47 (55.29%)	0.888
Vertigo (%)	13 (54.17%)	11 (45.85%)	0.300
Ear fullness (%)	15 (50%)	15 (50%)	0.511
Time to treatment (days)	7.00 (6.00, 8.00)	7.00 (6.00, 7.00)	0.680
HDL (mg/L FEU)	1.81 (1.27, 1.89)	1.58 (1.27, 1.88)	0.723
LDL (mmol/L)	2.65 (2.35, 3.74)	2.91 (2.42, 3.44)	0.843
HbA1C (mmol/L)	5.70 (5.20, 6.20)	5.70 (5.40, 6.20)	0.736
DD (mg/L FEU)	0.22 (0.14, 0.23)	0.18 (0.12, 0.24)	0.277
TBil (mg/dL)	10.60 (6.50, 16.0.64)	6.80 (6.20, 16.00)	0.166
Indirect_bilirubin (mg/dL)	9.00 (5.00, 13.60)	5.80 (3.60, 14.50)	0.358
Platelet_volume (×10^9^/L)	11.00 (10.20, 12.30)	11.40 (10.10, 12.50)	0.622
Creatinine (μmol/L)	69.00 (55.00, 84.50)	66.00 (55.00, 79.00)	0.591
AST (U/L)	18.00 (16.00, 22.50)	18.00 (16.00, 21.60)	0.615
ALT (U/L)	16.00 (10.00, 26.00)	21.00 (15.00, 26.00)	0.098
TG (umol/L)	0.62 (0.54, 1.00)	0.62 (0.55, 0.89)	0.871
TC (mmol/L)	4.21 (3.85, 4.82)	4.23 (3.88, 5.14)	0.698
FIB (g/L)	2.95 (2.43, 3.21)	2.99 (2.55, 3.76)	0.300
WBC (×10^9^/L)	8.10 (7.25, 9.08)	8.82 (6.21, 10.73)	0.808
ALB (g/L)	43.00 (36.00, 44.50)	43.00 (41.00, 45.00)	0.702
NE (×10^9^/L)	5.88 (2.90, 7.98)	6.34 (4.77, 7.87)	0.139
LYM (×10^9^/L)	1.64 (1.11, 2.61)	1.59 (1.25, 2.06)	0.547
PLT (×10^9^/L)	255.52 (216.90, 294.48)	257.75 (195.51, 277.97)	0.440
SII score (×10^9^/L)	650.06 (525.14, 904.27)	972.17 (714.26, 1138.15)	<0.001^*^
PNI score (%)	(53.32 ± 6.28)	(48.20 ± 7.21)	<0.001^*^
PTA (dBHL)	51.00 (41.50, 57.00)	63.00 (48.00, 90.00)	0.121
Post-treatment PTA (dBHL)	31.00 (22.50, 46.50)	62.00 (44.00, 81.00)	<0.001^*^
Mild to moderate (%)	33 (61.11%)	21 (38.89%)	<0.001^*^

SBP, admission systolic blood pressure; DBP, admission diastolic blood pressure; BMI, body mass index; HbA1c, glycosylated hemoglobin A1c; Cr, Creatinine, DD, D-D dimer; TC, total cholesterol; TG, triglycerides; HDL, high-density lipoprotein; LDL, low-density lipoprotein; FIB, Fibrinogen; WBC, white blood cell; ALB, albumin; AST, aspartate aminotransferase; ALT, alanine aminotransferase; TBIL, total bilirubin; IBIL, indirect bilirubin; FIB, fibrinogen; NE, Neutrophils; LYM, lymphocyte; PLT, platelet; PTA, pure-tone average; SSNHL, sudden sensorineural hearing loss. ^*^p < 0.05 (statistically significant).

### Correlation analysis between SII and PNI

**Figure 1:**
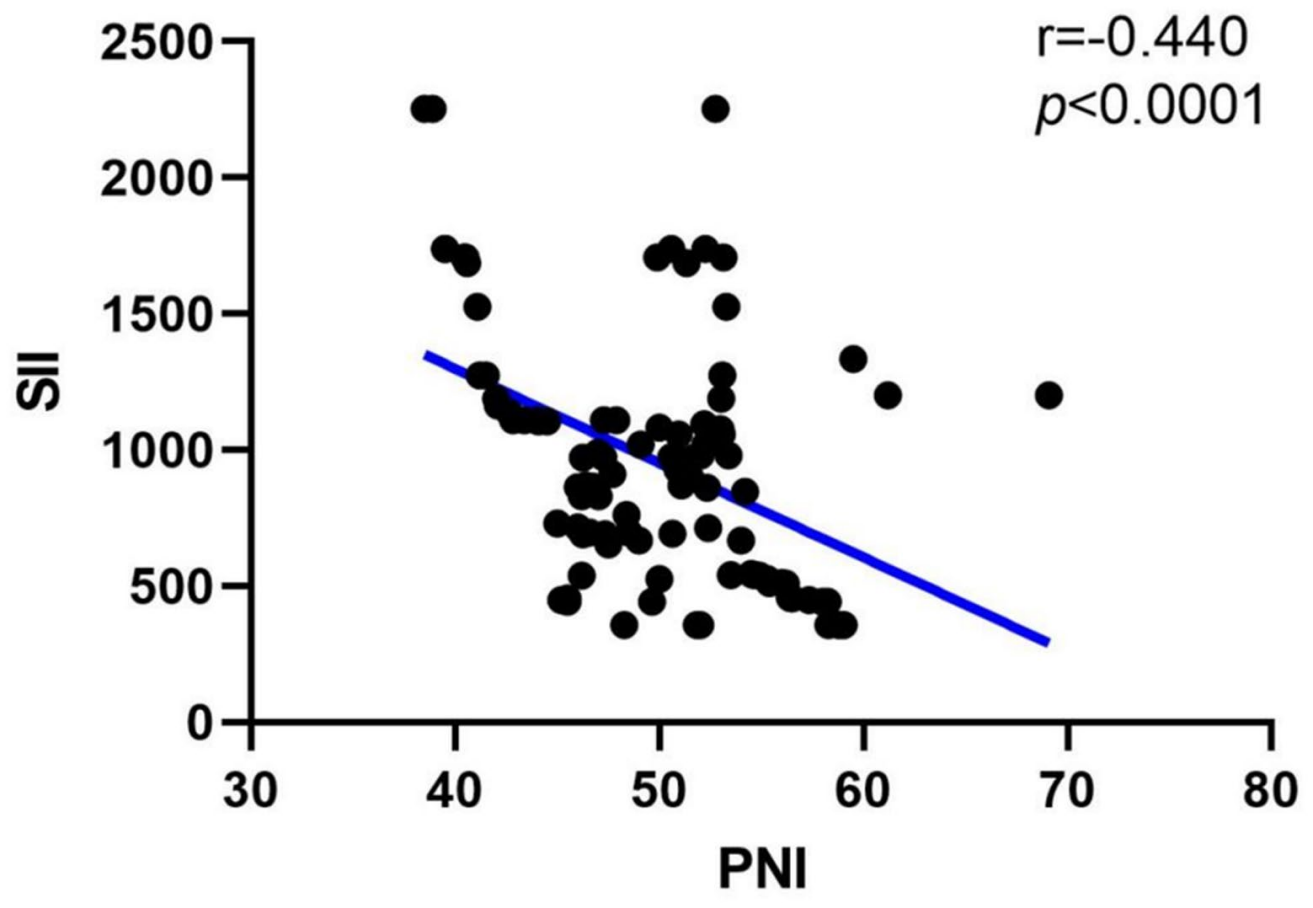
Correlation analysis between SII and PNI.

In order to observe the relationship between SII and PNI in SSNHL, we then performed a correlation analysis. The results were shown in Figure 1, *R* = −0.437, *p* < 0.001, and the above results indicated that there is a negative correlation between SII and PNI levels in the SSNHL group, and the correlation is statistically significant.

### Correlation analysis between SII, PNI and SSNHL symptoms

Further correlation analysis, summarized in Table 3, shows that SII and PNI correlate with pre-treatment PTA, post-treatment PTA, the coded severity of hearing loss (mild-to-moderate = 1; severe-to-profound = 2), and treatment effectiveness (effective = 1; ineffective = 0). Specifically, SII positively correlates with severity and negatively correlates with effectiveness. PNI negatively correlates with severity (once the severity code is aligned: 1 = mild-to-moderate, 2 = severe-to-profound) and positively correlates with treatment effectiveness.

**Table 3:** Correlations between the SII score and PNI score and SSNHL symptoms.

Parameters	SII		PNI	
	Spearman r	*p*-value	Spearman r	*p*-value
PTA	0.255	0.010	−0.261	0.009
Post-treatment PTA	0.395	0.010	−0.601	<0.001
Severity of hearing loss (1,2)	0.580	<0.001	−0.421	<0.001
Effective case (1,0)	−0.396	<0.001	0.383	<0.001

PTA, pure-tone average. *p* < 0.05 (statistically significant).

**Figure 2:**
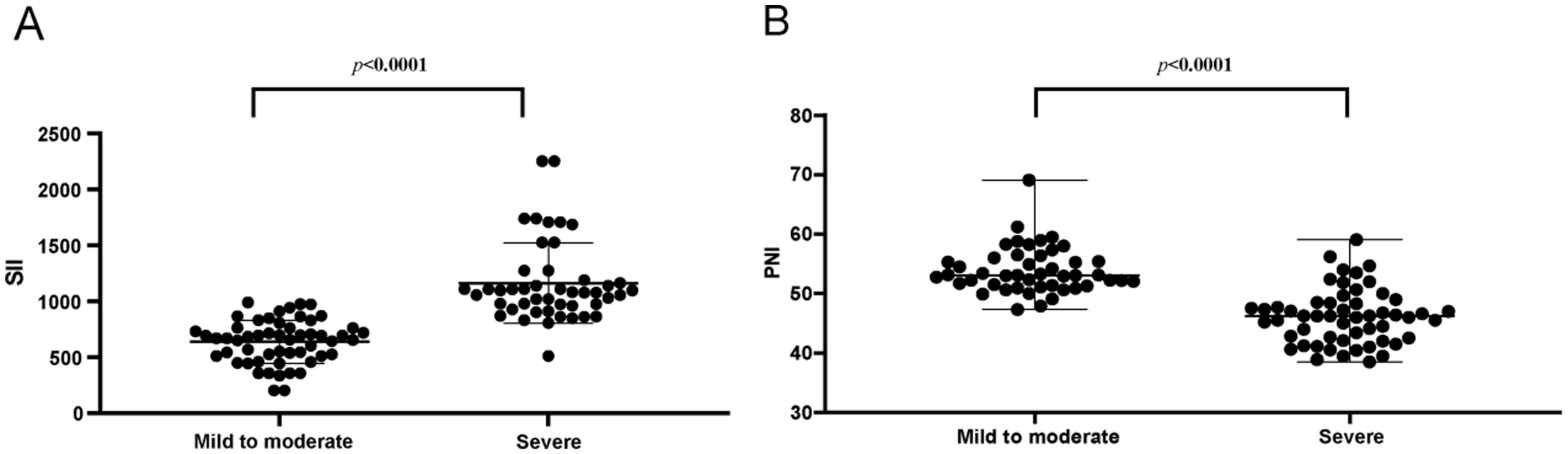
Relationships between severity of hearing loss and **(A)** SII and **(B)** PNI.

Secondly, the levels of SII and PNI were analyzed in patients exhibiting varying degrees of hearing loss severity. As illustrated in Figure 2, there was a statistically significant increase in SII levels and a concomitant significant decrease in PNI levels (*p* < 0.0001) in patients with severe hearing loss compared to those with mild and moderate hearing loss. This observed trend exhibited a clear gradient effect, indicating that SII and PNI levels may be correlated with the severity of sudden deafness.

### Relationship between SII and PNI and the prognosis of SSNHL

When combining all SSNHL patients into one model (as shown in Table 4), the adjusted odds ratio (OR) for SII was 0.912 (95% CI: 0.848–0.982, *p* = 0.014) and for PNI was 0.998 (95% CI: 0.996–0.999, *p* = 0.006). Given the coding of variables, the OR < 1 for SII and PNI reflects the inverse coding used for the effective case.

**Table 4:** Univariate and multivariate analysis in the overall effective and ineffective groups.

Variables	Univariate analysis		Multivariate analysis	
	*OR* (*95%CI*)	*p*	*OR* (*95%CI*)	*p*
Demographic, clinical, and metabolic variables				
Age (years)	1.011 (0.986, 1.036)	0.409		
Males (%)	1.500 (0.679, 3.312)	0.316		
BMI (kg/m^2^)	0.992 (0.889, 1.106)	0.879		
SBP	0.981 (0.945, 1.019)	0.320		
DBP	0.994 (0.936, 1.055)	0.833		
Affected side (%)	1.008 (0.458, 2.217)	0.984		
Tinnitus (%)	0.924 (0.307, 2.778)	0.888		
Vertigo (%)	2.429 (0.993, 5.939)	0.052		
Ear fullness (%)	1.330 (0.565, 3.145)	0.511		
Time to treatment	1.055 (0.848, 1.313)	0.629		
HDL (mg/L FEU)	1.387 (0.437, 4.404)	0.578		
LDL (mmol/L)	1.014 (0.676, 1.520)	0.946		
HbA1C	0.991 (0.625, 1.571)	0.969		
DD	1.655 (0.195, 14.016)	0.644		
TBil	1.034 (0.972, 1.100)	0.290		
Indirect_bilirubin	1.004 (0.933, 1.080)	0.912		
Platelet_volume	0.946 (0.733, 1.221)	0.670		
Creatinine	1.012 (0.984, 1.040)	0.409		
AST	1.033 (0.952, 1.120)	0.441		
ALT	0.979 (0.938, 1.021)	0.317		
TG (umol/L)	1.400 (0.488, 4.017)	0.532		
TC (mmol/L)	0.927 (0.610, 1.408)	0.722		
FIB	0.712 (0.386, 1.313)	0.277		
WBC	0.922 (0.778, 1.092)	0.348		
ALB	0.958 (0.878, 1.046)	0.338		
NE	0.865 (0.726, 1.031)	0.105		
LYM	0.984 (0.931, 1.039)	0.557		
PLT	1.004 (0.996, 1.012)	0.351		
SII score	0.998 (0.996, 0.999)	0.002^*^	0.912 (0.848, 0.982)	0.014^*^
PNI score	0.919 (0.853, 0.990)	0.026^*^	0.998 (0.996, 0.999)	0.006^*^

SBP, admission systolic blood pressure; DBP, admission diastolic blood pressure; BMI, body mass index; HbA1c, glycosylated hemoglobin A1c; Cr, Creatinine, DD, D-D dimer; TC, total cholesterol; TG, triglycerides; HDL, high-density lipoprotein; LDL, low-density lipoprotein; FIB, Fibrinogen; WBC, white blood cell; ALB, albumin; AST, aspartate aminotransferase; ALT, alanine aminotransferase; TBIL, total bilirubin; IBIL, indirect bilirubin; FIB, fibrinogen; NE, Neutrophils; LYM, lymphocyte; PLT, platelet; PTA, pure-tone average; SSNHL, sudden sensorineural hearing loss. ^*^p < 0.05 (statistically significant).

### Model validation of predictive

**Figure 3:**
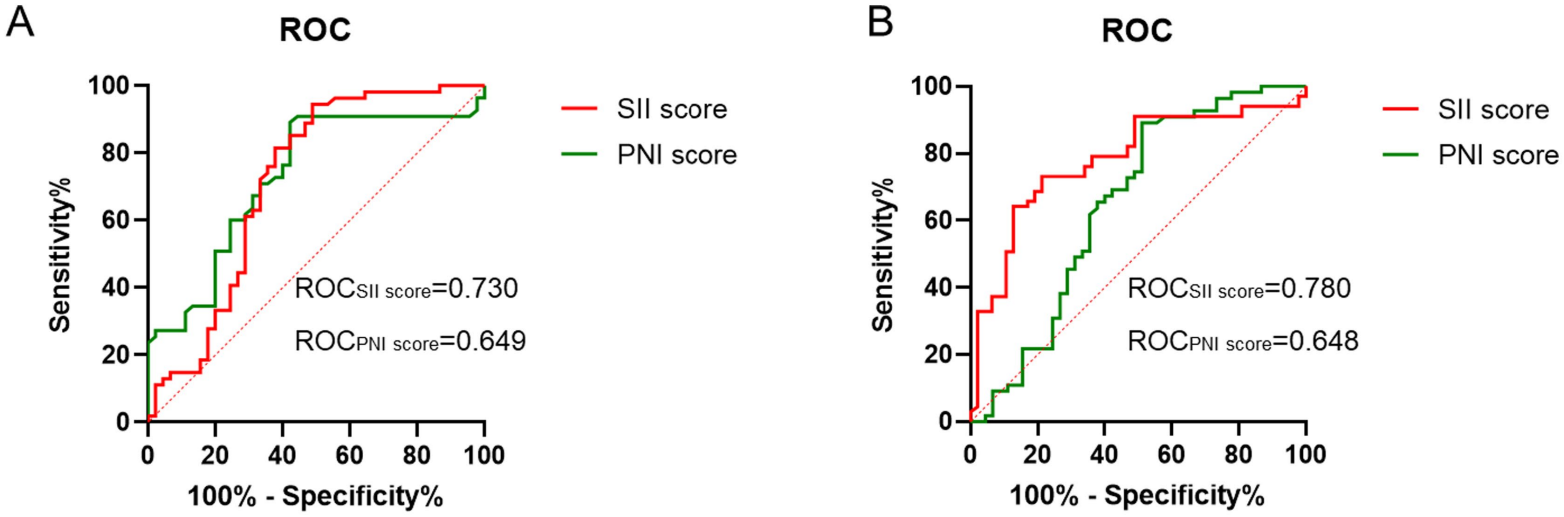
ROC curve analysis. **(A)** ROC curve analysis of PNI or SII for the prediction of the outcome of SSNHL. **(B)** ROC curve analysis of PNI or SII for the treatment outcome of SSNHL.

We used ROC curve analysis to assess the discriminative power of SII and PNI for distinguishing mild-to-moderate vs. severe-to-profound SSNHL. As shown in Figure 3, the area under the ROC curve (AUC) of SII was 0.730 (95% CI: 0.628–0.831, *p* < 0.001) and that of PNI was 0.649 (95% CI: 0.534–0.763, *p* = 0.007) (Figure 3A). Additional subgroup ROC analyses for effective vs. ineffective treatment indicated similar trends, the AUC of SII was 0.780 (95% CI: 0.693–0.867, *p* < 0.001) and that of PNI was 0.648 (95% CI: 0.534–0.763, *p* < 0.001) (Figure 3B), suggesting potential prognostic value in both severity categorization and therapeutic outcomes.

## Discussion

SSNHL is a common emergency in otolaryngology, characterized by sudden, unexplained hearing loss that can severely impact a patient’s quality of life. This study demonstrated that SSNHL patients exhibit a higher inflammatory status (elevated SII) and poorer nutritional/immune status (lower PNI) compared to healthy controls. A moderate to large effect size (Cohen’s d) in PNI underscores that these differences have practical and biological importance. Furthermore, higher SII and lower PNI correlated with more severe hearing loss and poorer hearing recovery, indicating that both biomarkers may be valuable indicators of SSNHL prognosis.

At the same time, SSNHL can also have a wide range of impacts on the patient’s psychology and quality of life (1). Early diagnosis and timely treatment are the key to restoring hearing for SSNHL patients (3). Screening for accurate diagnostic indicators is crucial for the early identification of patients, enabling the prompt implementation of effective treatment measures and enhancing the likelihood of hearing recovery. Consequently, the discovery of novel diagnostic indicators holds significant importance for improving the accuracy of SSNHL diagnosis, developing effective treatment protocols, assessing therapeutic outcomes, and advancing clinical research. Based on SII and PNI, we found that low PNI levels and high SII levels indicate more severe hearing loss and worse prognosis in SSNHL patients, and are potential diagnostic and prognostic indicators.

It is well known that the cause of SSNHL may be related to the inflammatory response of the inner ear, because the inner ear is a structure that is very sensitive to inflammation. Inflammation may cause damage or death of inner ear hair cells, leading to inner ear hair cell damage and hearing loss (15). In addition, for the inner ear, stable blood circulation is the guarantee for maintaining hearing function, and inflammatory response can lead to endothelial dysfunction. The increase of platelets and neutrophils and the aggregation and formation of thrombus may affect the blood supply of the inner ear (16). The progression of SSNHL was also involved in autoimmune reactions and oxidative stress, in which disordered immune responses and oxidative stress can mistakenly attack inner ear tissues, leading to hearing loss (17). The SII level is calculated by combining the platelet count, neutrophil count, and lymphocyte count. Neutrophils and platelets are important participants in the inflammatory response, while lymphocytes play a role in the anti-inflammatory process (7). SII can sensitively reflect the systemic inflammatory state by integrating these cell counts. Studies have shown that elevated SII is associated with a variety of diseases, including infection, autoimmune diseases and cancer (8, 9). High SII value reflect an increase in platelets and neutrophils, which may indicate increased blood viscosity and the risk of thrombosis. Moreover, high SII value also indicate an increase in neutrophils and a decrease in lymphocytes, which are related to disordered immune function. In short, the SII value can reflect the body’s systemic inflammatory state, inner ear microcirculation disorders, immune system disorders, and oxidative stress. By evaluating SII, doctors can better understand the inflammatory and immune status of SSNHL patients, thereby improving the accuracy of diagnosis, optimizing treatment plans, and predicting prognosis.

Compared to the control group, patients with SSNHL exhibited significantly elevated levels of SII, neutrophils, and platelets, alongside significantly reduced levels of PNI and albumin. Furthermore, within the SSNHL cohort, the effective group demonstrated a significantly higher SII level compared to the ineffective group, whereas the PNI level exhibited the opposite trend. Results of the correlation analysis between the PNI and SII indicated that they were negatively correlated in SSNHL. The above results showed that SSNHL patients have a higher inflammatory response and a poorer immune nutritional status, and higher inflammation and poorer immune nutritional status indicate a poor prognosis for SSNHL patients.

To our knowledge, PNI has never been reported as an indicator of the severity and prognosis of SSNHL. The health and function of inner ear hair cells require adequate nutritional support. Studies have shown that a lack of nutrients such as vitamin A, B vitamins, vitamin C, vitamin E, zinc, and iron may lead to hair cell damage and death, thereby affecting hearing. In addition, essential fatty acids and amino acids are important components of neurotransmitter synthesis (6). Malnutrition can result in inadequate synthesis of neurotransmitters, thereby impairing the transmission of auditory signals. Concurrently, the regeneration of damaged inner ear hair cells necessitates sufficient nutritional support, encompassing proteins, vitamins, and minerals (6). Therefore, malnutrition not only contributes to the incidence of SSNHL, but is also associated with the prognosis of affected patients. The PNI serves as a comprehensive indicator of nutritional and immune status, calculated using serum albumin levels and peripheral blood lymphocyte counts. Among these, albumin in plasma is a key marker for evaluating nutritional status and chronic inflammation. Low albumin levels are frequently observed in chronic diseases and malnutrition (18). SSNHL patients with good overall health usually respond better to treatment and are more likely to recover their hearing. It can be seen that PNI can help assess the patient’s overall health level, thereby predicting their response to treatment and prognosis. More importantly, malnutrition in patients often causes the occurrence of chronic diseases, including diabetes and cardiovascular disease, which may promote the occurrence of SSNHL and have an adverse effect on recovery after treatment (19). In addition, continuous monitoring of PNI can help doctors evaluate the effectiveness of treatment and adjust treatment plans in a timely manner to ensure that patients receive the best treatment results.

Therefore, by integrating SII and PNI, clinicians can better evaluate the inflammatory, immune, and nutritional status of SSNHL patients. This comprehensive approach can help inform treatment decisions, enabling more personalized care. Our study emphasizes the need for multidimensional intervention strategies in SSNHL management, such as anti-inflammatory therapy, nutritional support, immune enhancement, and lifestyle changes. For example, patients with very high SII and very low PNI may benefit from alternative treatments to avoid the side effects of corticosteroids. Intravenous hormone therapy (for 3 days), along with enhanced nutrition and physical activity, could help improve immune and nutritional status.

We also observed a significant correlation between SII and PNI levels and the degree of SSNHL hearing loss, suggesting that patients with long-term poor immunotrophic status may experience more severe hearing loss. This highlights the importance of closely monitoring the immune-nutritional status of SSNHL patients and taking proactive measures to manage it. Additionally, SII and PNI may serve as useful indicators to gauge the prognosis of SSNHL treatment, guiding clinicians in setting realistic goals and expectations, which can help improve patient confidence and treatment adherence.

Our study does present itself with several limitations. The relatively small sample size may lead to bias, and larger studies are needed to validate our findings. Furthermore, the absence of follow-up hearing assessments, and verified hearing parameters was a significant limitation, and future studies should include collecting more complete clinical data long-term and follow-up to assess the sustainability of treatment outcomes. Considering that the white blood cell counts of different individuals have different sensitivities to steroids, the bias caused by the effects of systemic methylprednisolone treatment on white blood cells and SII still exists. However, this bias does not undermine the final conclusion of this study.

## Conclusion

SII and PNI offer important prognostic information in SSNHL, with higher SII and lower PNI indicating a greater likelihood of severe hearing loss and unfavorable treatment outcomes. The moderate-to-large effect sizes for PNI differences highlight the practical relevance of these biomarkers. Monitoring SII and PNI may thus aid in early risk stratification, optimal therapeutic decision-making, and improved patient counseling. Future studies with larger cohorts and longer follow-up intervals are warranted to confirm and extend these findings.

## Data Availability

The raw data supporting the conclusions of this article will be made available by the authors, without undue reservation.
